# Supplementary material for: Specific labeling of synaptic schwann cells reveals unique cellular and molecular features
Source: eLife. 2020 Jun 25;9:e56935. doi: 10.7554/eLife.56935 (PMC7316509; doi:10.7554/eLife.56935)
Supplement: Supplementary file 1. [file elife-56935-supp1.docx]

**Supplementary File 1.** Genes identified in RNA seq analysis with a minimum copy count of 5 in PSCs that also display at least a four-fold increase in expression and a p-value of less than 0.05 in PSCs versus both S100B-GFP+ cells and NG2-dsRed+ cells. ND = not detected in cell type under comparison.

| **Gene** | **Description** | **Known Function in Synapse (s), PSC (p), or other Glia (g)?** | **Read Count** | **Log_2_ Fold Change vs S100B-GFP+** | **Log_2_ Fold Change vs NG2-dsRed+** |
| --- | --- | --- | --- | --- | --- |
| Adam11 | a disintegrin and metallopeptidase domain 11 |  | 505 | 4.43 | 4.22 |
| Adam12 | a disintegrin and metallopeptidase domain 12 (meltrin alpha) |  | 1209 | 3.63 | 4.49 |
| Adam23 | a disintegrin and metallopeptidase domain 23 |  | 2761 | 4.55 | 6.63 |
| Adamts20 | a disintegrin-like and metallopeptidase (reprolysin type) with thrombospondin type 1 motif, 20 |  | 382 | 2.72 | 4.87 |
| Asic4 | acid-sensing (proton-gated) ion channel family member 4 |  | 8 | 4.74 | 4.69 |
| Acsbg1 | acyl-CoA synthetase bubblegum family member 1 |  | 2619 | 6.59 | 8.48 |
| Acot1 | acyl-CoA thioesterase 1 |  | 173 | 2.49 | 2.82 |
| Adarb2 | adenosine deaminase, RNA-specific, B2 |  | 48 | 2.94 | 4.40 |
| Ajap1 | adherens junction associated protein 1 |  | 3172 | 7.97 | 5.77 |
| Adgrb1 | adhesion G protein-coupled receptor B1 | s | 86 | 3.99 | 6.12 |
| Adgrb3 | adhesion G protein-coupled receptor B3 | s | 69 | 2.37 | 4.90 |
| Adgrl3 | adhesion G protein-coupled receptor L3 | s | 1051 | 4.17 | 5.40 |
| Apba2 | amyloid beta (A4) precursor protein-binding, family A, member 2 | s | 98 | 4.28 | 7.12 |
| Anapc13 | anaphase promoting complex subunit 13 |  | 1519 | 2.50 | 2.45 |
| Adgb | androglobin |  | 31 | 2.31 | 4.82 |
| Angptl3 | angiopoietin-like 3 |  | 66 | 2.57 | 3.30 |
| Anks1b | ankyrin repeat and sterile alpha motif domain containing 1B | s | 291 | 4.77 | 6.23 |
| Aatk | apoptosis-associated tyrosine kinase |  | 1086 | 2.01 | 2.40 |
| Armh4 | armadillo-like helical domain containing 4 |  | 945 | 2.71 | 4.80 |
| Asrgl1 | asparaginase like 1 |  | 555 | 3.13 | 3.19 |
| Aspa | aspartoacylase | g | 1252 | 4.47 | 5.27 |
| Atp8a1 | ATPase, aminophospholipid transporter (APLT), class I, type 8A, member 1 |  | 1305 | 2.66 | 2.33 |
| Abca8b | ATP-binding cassette, sub-family A (ABC1), member 8b |  | 2557 | 3.49 | 2.95 |
| Bhlhe22 | basic helix-loop-helix family, member e22 |  | 37 | 3.04 | 2.54 |
| Bmp6 | bone morphogenetic protein 6 | g | 1319 | 2.70 | 2.48 |
| Bex1 | brain expressed X-linked 1 |  | 20 | 2.67 | 3.17 |
| Bex4 | brain expressed X-linked 4 |  | 52 | 5.13 | 4.64 |
| Bche | butyrylcholinesterase | p,s | 7191 | 7.21 | 7.89 |
| C2cd4d | C2 calcium-dependent domain containing 4D |  | 18 | 3.65 | 4.42 |
| Cdh10 | cadherin 10 | s | 194 | 5.09 | 6.88 |
| Cdh19 | cadherin 19, type 2 |  | 1931 | 4.98 | 5.12 |
| Cdh20 | cadherin 20 |  | 49 | 4.08 | 5.38 |
| Celsr1 | cadherin, EGF LAG seven-pass G-type receptor 1 |  | 126 | 3.20 | 4.11 |
| Celsr2 | cadherin, EGF LAG seven-pass G-type receptor 2 |  | 223 | 2.70 | 3.94 |
| Cacng5 | calcium channel, voltage-dependent, gamma subunit 5 |  | 95 | 4.60 | 3.86 |
| Camk2b | calcium/calmodulin-dependent protein kinase II, beta | g,s | 649 | 4.53 | 5.09 |
| Car12 | carbonic anhydrase 12 |  | 1762 | 6.10 | 7.37 |
| Cpa2 | carboxypeptidase A2, pancreatic |  | 15 | 2.59 | 3.30 |
| Cpm | carboxypeptidase M |  | 12914 | 7.20 | 3.91 |
| Ctnnal1 | catenin (cadherin associated protein), alpha-like 1 |  | 1795 | 3.05 | 4.77 |
| Cd59a | CD59a antigen | g | 1172 | 3.31 | 2.32 |
| Cd59b | CD59b antigen |  | 74 | 3.42 | 2.86 |
| Arhgef9 | CDC42 guanine nucleotide exchange factor (GEF) 9 | s | 369 | 3.40 | 2.55 |
| BC064078 | cDNA sequence BC064078 |  | 161 | 2.55 | 4.86 |
| BC106179 | cDNA sequence BC106179 |  | 54 | 3.03 | 3.35 |
| Cadm1 | cell adhesion molecule 1 | g,s | 3177 | 4.40 | 6.32 |
| Cadm2 | cell adhesion molecule 2 |  | 115 | 2.69 | 4.54 |
| Cadm4 | cell adhesion molecule 4 | g | 1388 | 4.08 | 6.20 |
| Chl1 | cell adhesion molecule L1-like |  | 3637 | 5.61 | 7.60 |
| Cenpw | centromere protein W |  | 109 | 2.53 | 2.91 |
| Chadl | chondroadherin-like |  | 360 | 3.07 | 4.46 |
| Cspg5 | chondroitin sulfate proteoglycan 5 | s | 240 | 3.83 | 3.98 |
| Cbx3-ps7 | chromobox 3, pseudogene 7 |  | 44 | 3.43 | 2.36 |
| Cela1 | chymotrypsin-like elastase family, member 1 |  | 42 | 4.00 | 4.67 |
| Cmtm5 | CKLF-like MARVEL transmembrane domain containing 5 |  | 1267 | 4.34 | 6.78 |
| Cldn11 | claudin 11 | g | 50 | 2.42 | 3.05 |
| Clvs1 | clavesin 1 |  | 132 | 4.71 | 6.12 |
| Cdrt4os1 | CMT1A duplicated region transcript 4, opposite strand 1 |  | 27 | 4.49 | 5.11 |
| Ccdc13 | coiled-coil domain containing 13 |  | 89 | 2.66 | 4.87 |
| Ccdc30 | coiled-coil domain containing 30 | g | 97 | 2.41 | 4.17 |
| Col4a4 | collagen, type IV, alpha 4 |  | 553 | 2.30 | 2.52 |
| Col9a2 | collagen, type IX, alpha 2 |  | 258 | 4.16 | 3.71 |
| Col9a3 | collagen, type IX, alpha 3 |  | 573 | 5.06 | 7.15 |
| Col11a1 | collagen, type XI, alpha 1 |  | 1883 | 2.93 | 3.27 |
| Col20a1 | collagen, type XX, alpha 1 |  | 11021 | 7.50 | 7.92 |
| Col27a1 | collagen, type XXVII, alpha 1 |  | 1765 | 4.01 | 3.90 |
| C1ql1 | complement component 1, q subcomponent-like 1 |  | 214 | 7.13 | 7.40 |
| Cnksr2 | connector enhancer of kinase suppressor of Ras 2 |  | 174 | 3.87 | 2.52 |
| Cntn6 | contactin 6 |  | 74 | 3.49 | 6.82 |
| Ctxn1 | cortexin 1 |  | 134 | 2.35 | 2.06 |
| Cryab | crystallin, alpha B |  | 3407 | 2.33 | 2.34 |
| Cryl1 | crystallin, lambda 1 |  | 1138 | 3.53 | 4.23 |
| Crym | crystallin, mu |  | 304 | 4.43 | 5.12 |
| Clec14a | C-type lectin domain family 14, member a |  | 1502 | 3.93 | 2.42 |
| Csmd1 | CUB and Sushi multiple domains 1 |  | 619 | 3.99 | 7.16 |
| Csmd3 | CUB and Sushi multiple domains 3 |  | 201 | 4.09 | 7.58 |
| Ccnd1 | cyclin D1 |  | 648 | 2.70 | 2.23 |
| Cntd1 | cyclin N-terminal domain containing 1 |  | 14 | 2.22 | 2.69 |
| Cyp2j6 | cytochrome P450, family 2, subfamily j, polypeptide 6 |  | 1389 | 3.40 | 3.62 |
| Cyp2j9 | cytochrome P450, family 2, subfamily j, polypeptide 9 |  | 1347 | 4.51 | 5.12 |
| Ckap2 | cytoskeleton associated protein 2 |  | 480 | 2.27 | 2.78 |
| Ddx43 | DEAD (Asp-Glu-Ala-Asp) box polypeptide 43 |  | 39 | 4.62 | 4.33 |
| Defb25 | defensin beta 25 |  | 25 | 2.28 | 2.19 |
| Dhrs2 | dehydrogenase/reductase member 2 |  | 345 | 6.63 | 8.10 |
| Depdc7 | DEP domain containing 7 |  | 412 | 3.37 | 5.81 |
| Dagla | diacylglycerol lipase, alpha |  | 249 | 2.20 | 3.70 |
| Dbi | diazepam binding inhibitor | g | 13823 | 3.44 | 4.33 |
| Dpyd | dihydropyrimidine dehydrogenase |  | 371 | 3.33 | 4.56 |
| Dab1 | disabled 1 | g | 68 | 3.90 | 4.67 |
| Dlgap1 | DLG associated protein 1 | s | 412 | 3.67 | 5.55 |
| Dct | dopachrome tautomerase |  | 427 | 7.46 | 9.81 |
| Dbh | dopamine beta hydroxylase | s | 75 | 4.21 | 7.66 |
| Dnm3 | dynamin 3 | s | 724 | 3.44 | 2.18 |
| Dynlrb2 | dynein light chain roadblock-type 2 |  | 5 | 3.21 | ND |
| Dnaic2 | dynein, axonemal, intermediate chain 2 |  | 121 | 3.19 | 4.15 |
| Dtna | dystrobrevin alpha | g,s | 247 | 2.13 | 2.14 |
| Dag1 | dystroglycan 1 | g,s | 20491 | 3.39 | 3.07 |
| Egfem1 | EGF-like and EMI domain containing 1 |  | 56 | 3.47 | 2.17 |
| Egfl8 | EGF-like domain 8 |  | 749 | 2.44 | 4.55 |
| Elovl2 | elongation of very long chain fatty acids (FEN1/Elo2, SUR4/Elo3, yeast)-like 2 |  | 26 | 2.49 | 6.90 |
| Eno4 | enolase 4 |  | 14 | 2.11 | 3.41 |
| Erbb3 | erb-b2 receptor tyrosine kinase 3 | g,p | 2471 | 4.46 | 7.05 |
| Epb41l4b | erythrocyte membrane protein band 4.1 like 4b |  | 1606 | 5.12 | 6.60 |
| Etv1 | ets variant 1 |  | 2431 | 4.99 | 2.65 |
| Etv5 | ets variant 5 | s | 1068 | 3.52 | 2.68 |
| AI197445 | expressed sequence AI197445 |  | 16 | 2.01 | 3.23 |
| Fam102a | family with sequence similarity 102, member A |  | 538 | 2.32 | 2.02 |
| Fam161b | family with sequence similarity 161, member B |  | 24 | 2.38 | 2.00 |
| Fam181b | family with sequence similarity 181, member B |  | 292 | 4.21 | 2.02 |
| Fam184a | family with sequence similarity 184, member A |  | 217 | 3.61 | 4.04 |
| Fam184b | family with sequence similarity 184, member B |  | 316 | 4.81 | 6.41 |
| Fabp7 | fatty acid binding protein 7, brain |  | 721 | 4.60 | 6.86 |
| Fbxw7 | F-box and WD-40 domain protein 7 |  | 980 | 2.52 | 2.75 |
| Fbxo44 | F-box protein 44 |  | 63 | 2.82 | 3.04 |
| Fibp | fibroblast growth factor (acidic) intracellular binding protein |  | 1254 | 2.73 | 2.53 |
| Fign | fidgetin | g | 445 | 3.66 | 4.70 |
| Fibin | fin bud initiation factor homolog (zebrafish) |  | 1639 | 4.73 | 4.61 |
| Foxd3 | forkhead box D3 |  | 1760 | 5.20 | 7.72 |
| Fzd1 | frizzled class receptor 1 |  | 1986 | 3.10 | 4.45 |
| Fbp1 | fructose bisphosphatase 1 |  | 25 | 3.73 | 5.43 |
| Fxyd1 | FXYD domain-containing ion transport regulator 1 |  | 9201 | 3.71 | 3.16 |
| Fxyd3 | FXYD domain-containing ion transport regulator 3 |  | 325 | 2.33 | 4.82 |
| Fxyd7 | FXYD domain-containing ion transport regulator 7 |  | 67 | 4.67 | 4.14 |
| Gpr156 | G protein-coupled receptor 156 |  | 18 | 2.43 | 3.98 |
| Gpr17 | G protein-coupled receptor 17 | g | 147 | 4.38 | 4.68 |
| Gpr37l1 | G protein-coupled receptor 37-like 1 | g | 2891 | 5.19 | 6.87 |
| Gal3st1 | galactose-3-O-sulfotransferase 1 | g | 480 | 3.23 | 6.07 |
| Gabra1 | gamma-aminobutyric acid (GABA) A receptor, subunit alpha 1 | s | 89 | 4.51 | 6.47 |
| Ggt7 | gamma-glutamyltransferase 7 |  | 71 | 2.87 | 2.05 |
| Gjc3 | gap junction protein, gamma 3 | g | 3609 | 3.30 | 6.19 |
| Glis3 | GLIS family zinc finger 3 |  | 473 | 2.89 | 4.94 |
| Gria3 | glutamate receptor, ionotropic, AMPA3 (alpha 3) | s | 221 | 2.15 | 2.79 |
| Gria4 | glutamate receptor, ionotropic, AMPA4 (alpha 4) | s | 118 | 2.01 | 4.58 |
| Grik2 | glutamate receptor, ionotropic, kainate 2 (beta 2) | s | 448 | 4.98 | 7.64 |
| Grik3 | glutamate receptor, ionotropic, kainate 3 | s | 37 | 2.70 | 3.42 |
| Grm5 | glutamate receptor, metabotropic 5 | p,s | 38 | 2.84 | 6.64 |
| Gpt2 | glutamic pyruvate transaminase (alanine aminotransferase) 2 |  | 1116 | 4.50 | 4.85 |
| Gstm6 | glutathione S-transferase, mu 6 |  | 41 | 2.34 | 3.09 |
| Gdpd2 | glycerophosphodiester phosphodiesterase domain containing 2 |  | 10 | 2.28 | 2.45 |
| Gpm6b | glycoprotein m6b | g | 6853 | 3.80 | 5.72 |
| Gramd1c | GRAM domain containing 1C |  | 66 | 2.52 | 3.59 |
| Gas2l3 | growth arrest-specific 2 like 3 |  | 1132 | 2.02 | 4.94 |
| H1fx | H1 histone family, member X |  | 97 | 2.37 | 2.06 |
| Hspa12a | heat shock protein 12A |  | 2429 | 3.49 | 2.88 |
| Hexim2 | hexamethylene bis-acetamide inducible 2 |  | 97 | 3.73 | 3.21 |
| Hmgb2 | high mobility group box 2 |  | 2401 | 2.61 | 2.81 |
| Hist1h2ab | histone cluster 1, H2ab |  | 49 | 2.10 | 3.18 |
| Hist1h2ae | histone cluster 1, H2ae |  | 210 | 2.72 | 4.11 |
| Hist1h2an | histone cluster 1, H2an |  | 16 | 2.42 | 4.37 |
| Hist1h2ao | histone cluster 1, H2ao |  | 511 | 2.62 | 3.50 |
| Hist1h2ap | histone cluster 1, H2ap |  | 647 | 2.76 | 3.59 |
| Hist1h3i | histone cluster 1, H3i |  | 67 | 2.22 | 3.09 |
| Hist1h4d | histone cluster 1, H4d |  | 3364 | 3.09 | 2.70 |
| Hoxb5os | homeobox B5 and homeobox B6, opposite strand |  | 24 | 5.05 | 2.48 |
| Hunk | hormonally upregulated Neu-associated kinase |  | 187 | 3.96 | 3.49 |
| Hsd17b11 | hydroxysteroid (17-beta) dehydrogenase 11 |  | 1230 | 2.45 | 2.19 |
| Igsf11 | immunoglobulin superfamily, member 11 |  | 667 | 4.55 | 7.09 |
| Igsf9b | immunoglobulin superfamily, member 9B | s | 1480 | 5.01 | 4.60 |
| Inka2 | inka box actin regulator 2 |  | 698 | 3.70 | 2.46 |
| Inava | innate immunity activator |  | 13 | 2.87 | 4.07 |
| Insc | INSC spindle orientation adaptor protein |  | 210 | 2.85 | 2.46 |
| Insl6 | insulin-like 6 |  | 19 | 2.49 | 3.25 |
| Itga2 | integrin alpha 2 |  | 664 | 2.16 | 4.65 |
| Itgb8 | integrin beta 8 | g | 883 | 2.62 | 4.50 |
| Il1rap | interleukin 1 receptor accessory protein |  | 1317 | 3.03 | 3.37 |
| Il1rapl1 | interleukin 1 receptor accessory protein-like 1 | s | 144 | 3.80 | 6.17 |
| Josd2 | Josephin domain containing 2 |  | 506 | 2.24 | 2.44 |
| Klk13 | kallikrein related-peptidase 13 |  | 14 | 2.59 | 5.32 |
| Klk8 | kallikrein related-peptidase 8 | g,s | 283 | 4.89 | 4.01 |
| Klk9 | kallikrein related-peptidase 9 |  | 17 | 4.13 | 3.99 |
| Klhl34 | kelch-like 34 |  | 33 | 3.62 | 4.83 |
| Krtap7-1 | keratin associated protein 7-1 |  | 7 | 3.93 | ND |
| Kif21a | kinesin family member 21A |  | 860 | 2.88 | 3.81 |
| Kank4 | KN motif and ankyrin repeat domains 4 |  | 4659 | 5.92 | 5.22 |
| Kank4os | KN motif and ankyrin repeat domains 4, opposite strand |  | 38 | 4.49 | 4.59 |
| L1cam | L1 cell adhesion molecule | g,s | 2035 | 4.42 | 6.50 |
| Lrat | lecithin-retinol acyltransferase (phosphatidylcholine-retinol-O-acyltransferase) |  | 26 | 2.80 | 4.14 |
| Lrrc4b | leucine rich repeat containing 4B | s | 249 | 4.82 | 6.07 |
| Lrrc4c | leucine rich repeat containing 4C | s | 230 | 4.71 | 2.26 |
| Lrrc75b | leucine rich repeat containing 75B |  | 169 | 3.87 | 4.63 |
| Lrrn3 | leucine rich repeat protein 3, neuronal | s | 133 | 3.72 | 5.00 |
| Lrrtm1 | leucine rich repeat transmembrane neuronal 1 | s | 97 | 2.68 | 5.20 |
| Lrrtm4 | leucine rich repeat transmembrane neuronal 4 | s | 20 | 2.48 | 4.27 |
| Luzp2 | leucine zipper protein 2 |  | 512 | 5.34 | 7.08 |
| Lgi4 | leucine-rich repeat LGI family, member 4 | g | 2270 | 4.46 | 5.37 |
| Lhfpl2 | lipoma HMGIC fusion partner-like 2 |  | 1434 | 2.20 | 2.35 |
| Lhfpl4 | lipoma HMGIC fusion partner-like protein 4 |  | 44 | 2.34 | 2.61 |
| Lockd | lncRNA downstream of Cdkn1b |  | 662 | 3.85 | 4.20 |
| Lsm7 | LSM7 homolog, U6 small nuclear RNA and mRNA degradation associated |  | 495 | 2.48 | 2.28 |
| Lhcgr | luteinizing hormone/choriogonadotropin receptor |  | 39 | 4.71 | 3.98 |
| Lypd6 | LY6/PLAUR domain containing 6 |  | 273 | 4.34 | 5.00 |
| Ly6g6d | lymphocyte antigen 6 complex, locus G6D |  | 13 | 2.22 | 2.53 |
| Ly6g6f | lymphocyte antigen 6 complex, locus G6F |  | 85 | 6.05 | 8.60 |
| Kdm4d | lysine (K)-specific demethylase 4D |  | 15 | 2.66 | 3.02 |
| Lpcat2 | lysophosphatidylcholine acyltransferase 2 |  | 382 | 2.47 | 5.78 |
| Mro | maestro |  | 20 | 2.67 | 4.64 |
| Mkrn3 | makorin, ring finger protein, 3 |  | 18 | 3.00 | 2.59 |
| Mamdc2 | MAM domain containing 2 |  | 1050 | 3.02 | 2.18 |
| Mdga2 | MAM domain containing glycosylphosphatidylinositol anchor 2 |  | 128 | 3.70 | 4.11 |
| Matn2 | matrilin 2 | g | 7801 | 4.20 | 2.70 |
| Matn4 | matrilin 4 |  | 1402 | 4.35 | 6.57 |
| Mmp16 | matrix metallopeptidase 16 |  | 448 | 2.85 | 3.61 |
| Mmp17 | matrix metallopeptidase 17 |  | 686 | 4.39 | 2.68 |
| Mxd3 | Max dimerization protein 3 |  | 99 | 2.12 | 2.61 |
| Med9os | mediator complex subunit 9, opposite strand |  | 19 | 3.70 | 2.25 |
| Mns1 | meiosis-specific nuclear structural protein 1 |  | 134 | 3.13 | 3.38 |
| Mpp7 | membrane protein, palmitoylated 7 (MAGUK p55 subfamily member 7) |  | 351 | 2.10 | 2.26 |
| Metrn | meteorin, glial cell differentiation regulator | g | 158 | 4.14 | 4.05 |
| Mbd4 | methyl-CpG binding domain protein 4 |  | 171 | 2.55 | 2.04 |
| Micall2 | MICAL-like 2 |  | 359 | 3.78 | 2.69 |
| Map2 | microtubule-associated protein 2 |  | 656 | 4.42 | 2.25 |
| Map3k4 | mitogen-activated protein kinase kinase kinase 4 |  | 862 | 2.30 | 2.35 |
| Mok | MOK protein kinase |  | 30 | 2.21 | 2.41 |
| Morn4 | MORN repeat containing 4 |  | 56 | 3.34 | 3.63 |
| Megf10 | multiple EGF-like-domains 10 |  | 792 | 4.79 | 4.59 |
| Megf9 | multiple EGF-like-domains 9 |  | 3048 | 2.33 | 4.20 |
| Myh14 | myosin, heavy polypeptide 14 |  | 198 | 3.22 | 3.71 |
| Myh6 | myosin, heavy polypeptide 6, cardiac muscle, alpha |  | 33 | 2.58 | 3.93 |
| Nkain2 | Na+/K+ transporting ATPase interacting 2 |  | 262 | 3.91 | 6.86 |
| Nkain4 | Na+/K+ transporting ATPase interacting 4 |  | 613 | 5.01 | 5.79 |
| Nat8f1 | N-acetyltransferase 8 (GCN5-related) family member 1 |  | 156 | 2.64 | 2.50 |
| Nanos3 | nanos C2HC-type zinc finger 3 |  | 52 | 4.34 | 3.21 |
| Ndst3 | N-deacetylase/N-sulfotransferase (heparan glucosaminyl) 3 |  | 167 | 4.70 | 2.98 |
| Nell2 | NEL-like 2 |  | 22 | 2.13 | 4.82 |
| Ntng1 | netrin G1 | s | 982 | 5.56 | 4.95 |
| Ncam1 | neural cell adhesion molecule 1 | g,s | 3976 | 5.00 | 5.55 |
| Ncam2 | neural cell adhesion molecule 2 |  | 261 | 5.09 | 6.24 |
| Nrxn1 | neurexin I | s | 2269 | 6.59 | 7.68 |
| Nrxn3 | neurexin III | s | 176 | 3.53 | 5.23 |
| Nxph1 | neurexophilin 1 |  | 40 | 3.75 | 6.68 |
| Nrn1 | neuritin 1 | s | 305 | 5.09 | 6.51 |
| Nlgn1 | neuroligin 1 | g,s | 60 | 2.72 | 6.52 |
| Nlgn3 | neuroligin 3 | g,s | 390 | 5.30 | 5.51 |
| Nsg2 | neuron specific gene family member 2 |  | 232 | 5.96 | 7.01 |
| Negr1 | neuronal growth regulator 1 |  | 921 | 3.74 | 5.90 |
| Nptx1 | neuronal pentraxin 1 | s | 36 | 2.03 | 3.18 |
| Nnat | neuronatin |  | 103 | 2.15 | 3.98 |
| Npb | neuropeptide B |  | 12 | 3.37 | 4.29 |
| Neto2 | neuropilin (NRP) and tolloid (TLL)-like 2 |  | 189 | 3.09 | 2.85 |
| Nkx2-2 | NK2 homeobox 2 | g | 71 | 4.84 | 6.80 |
| Nkx2-2os | NK2 homeobox 2, opposite strand | g | 30 | 3.31 | 7.19 |
| Nme5 | NME/NM23 family member 5 |  | 32 | 3.28 | 3.15 |
| Nfatc2 | nuclear factor of activated T cells, cytoplasmic, calcineurin dependent 2 |  | 1371 | 2.54 | 3.55 |
| Nudt10 | nudix (nucleoside diphosphate linked moiety X)-type motif 10 |  | 16 | 2.70 | 2.34 |
| Olfr889 | olfactory receptor 889 |  | 26 | 2.47 | 3.92 |
| Pnlip | pancreatic lipase |  | 20 | 3.41 | 6.27 |
| Pth2r | parathyroid hormone 2 receptor |  | 131 | 5.61 | 7.63 |
| Pacrg | PARK2 co-regulated |  | 35 | 2.28 | 4.29 |
| Pdlim4 | PDZ and LIM domain 4 |  | 4298 | 4.08 | 4.32 |
| Pbk | PDZ binding kinase |  | 216 | 2.07 | 2.85 |
| Pdzrn4 | PDZ domain containing RING finger 4 |  | 82 | 3.76 | 5.94 |
| Pex11a | peroxisomal biogenesis factor 11 alpha |  | 61 | 2.08 | 4.60 |
| Pex5l | peroxisomal biogenesis factor 5-like |  | 310 | 4.45 | 4.01 |
| Pcyt1b | phosphate cytidylyltransferase 1, choline, beta isoform |  | 136 | 3.52 | 5.44 |
| Prex1 | phosphatidylinositol-3,4,5-trisphosphate-dependent Rac exchange factor 1 | s | 2281 | 2.50 | 4.44 |
| Pde4d | phosphodiesterase 4D, cAMP specific |  | 348 | 2.64 | 2.50 |
| Plppr1 | phospholipid phosphatase related 1 |  | 21 | 2.81 | 7.52 |
| Phyhipl | phytanoyl-CoA hydroxylase interacting protein-like |  | 122 | 3.91 | 5.26 |
| Pih1d2 | PIH1 domain containing 2 |  | 19 | 2.87 | 2.16 |
| Pdgfa | platelet derived growth factor, alpha | g | 5205 | 5.25 | 3.91 |
| Plekhb1 | pleckstrin homology domain containing, family B (evectins) member 1 |  | 2519 | 2.84 | 4.75 |
| Ptn | pleiotrophin | g,s | 7877 | 3.64 | 5.10 |
| Plxnb3 | plexin B3 |  | 879 | 3.61 | 6.23 |
| Poc1a | POC1 centriolar protein A |  | 90 | 2.44 | 2.97 |
| Paip2b | poly(A) binding protein interacting protein 2B |  | 716 | 2.21 | 2.07 |
| Kcnk10 | potassium channel, subfamily K, member 10 |  | 78 | 3.37 | 7.25 |
| Kcnn2 | potassium intermediate/small conductance calcium-activated channel, subfamily N, member 2 | s | 283 | 5.67 | 6.71 |
| Kcnj10 | potassium inwardly-rectifying channel, subfamily J, member 10 | g | 590 | 3.28 | 7.09 |
| Kcnj3 | potassium inwardly-rectifying channel, subfamily J, member 3 |  | 14 | 3.12 | ND |
| Kcnmb4 | potassium large conductance calcium-activated channel, subfamily M, beta member 4 | s | 391 | 4.53 | 5.07 |
| Kcnmb4os2 | potassium large conductance calcium-activated channel, subfamily M, beta member 4, opposite strand 2 |  | 31 | 3.07 | 6.02 |
| Kcna1 | potassium voltage-gated channel, shaker-related subfamily, member 1 | s | 2621 | 2.92 | 5.66 |
| Kcna2 | potassium voltage-gated channel, shaker-related subfamily, member 2 |  | 3927 | 3.94 | 5.91 |
| Kcna6 | potassium voltage-gated channel, shaker-related, subfamily, member 6 |  | 798 | 4.94 | 5.84 |
| Kcnh8 | potassium voltage-gated channel, subfamily H (eag-related), member 8 |  | 321 | 5.64 | 7.11 |
| Kcnq5 | potassium voltage-gated channel, subfamily Q, member 5 |  | 69 | 3.14 | 3.71 |
| Pou3f1 | POU domain, class 3, transcription factor 1 | g | 7220 | 4.76 | 6.92 |
| Pou3f2 | POU domain, class 3, transcription factor 2 | g | 113 | 3.39 | 6.01 |
| Pou3f4 | POU domain, class 3, transcription factor 4 |  | 59 | 4.17 | 5.43 |
| Prdm16os | Prdm16 opposite strand transcript |  | 150 | 4.51 | 3.19 |
| Pbx4 | pre B cell leukemia homeobox 4 |  | 19 | 2.08 | 2.22 |
| Gm10046 | predicted gene 10046 |  | 49 | 4.44 | 4.92 |
| Gm10146 | predicted gene 10146 |  | 160 | 2.70 | 2.48 |
| Gm10544 | predicted gene 10544 |  | 77 | 4.45 | 4.26 |
| Gm10558 | predicted gene 10558 |  | 34 | 3.92 | 3.23 |
| Gm10561 | predicted gene 10561 |  | 22 | 2.44 | 2.15 |
| Gm10657 | predicted gene 10657 |  | 18 | 2.39 | 3.11 |
| Gm10863 | predicted gene 10863 |  | 166 | 3.85 | 5.30 |
| Gm10941 | predicted gene 10941 |  | 27 | 2.45 | 2.22 |
| Gm11149 | predicted gene 11149 |  | 64 | 4.24 | 4.54 |
| Gm11266 | predicted gene 11266 |  | 37 | 2.45 | 3.05 |
| Gm11611 | predicted gene 11611 |  | 11 | 5.82 | 4.40 |
| Gm11697 | predicted gene 11697 |  | 6 | 5.39 | 4.15 |
| Gm11734 | predicted gene 11734 |  | 16 | 3.55 | 3.51 |
| Gm11816 | predicted gene 11816 |  | 137 | 4.07 | 3.98 |
| Gm12128 | predicted gene 12128 |  | 11 | 3.10 | ND |
| Gm12222 | predicted gene 12222 |  | 21 | 2.54 | 3.37 |
| Gm12530 | predicted gene 12530 |  | 21 | 3.17 | 5.33 |
| Gm12688 | predicted gene 12688 |  | 594 | 6.09 | 8.32 |
| Gm12705 | predicted gene 12705 |  | 11 | 3.88 | 2.15 |
| Gm12829 | predicted gene 12829 |  | 6 | 3.10 | 2.95 |
| Gm12851 | predicted gene 12851 |  | 9 | ND | 5.79 |
| Gm12976 | predicted gene 12976 |  | 7 | 3.84 | 3.81 |
| Gm13133 | predicted gene 13133 |  | 29 | 5.32 | 5.21 |
| Gm13174 | predicted gene 13174 |  | 75 | 6.42 | 7.95 |
| Gm13175 | predicted gene 13175 |  | 10 | 3.40 | 2.90 |
| Gm13187 | predicted gene 13187 |  | 65 | 4.80 | 4.37 |
| Gm13237 | predicted gene 13237 |  | 36 | 2.71 | 3.19 |
| Gm13403 | predicted gene 13403 |  | 48 | 3.33 | 4.92 |
| Gm13479 | predicted gene 13479 |  | 21 | 2.27 | 5.35 |
| Gm13491 | predicted gene 13491 |  | 22 | 3.65 | 5.66 |
| Gm13830 | predicted gene 13830 |  | 22 | 3.06 | 2.57 |
| Gm13963 | predicted gene 13963 |  | 9 | 2.62 | ND |
| Gm13967 | predicted gene 13967 |  | 8 | 5.73 | ND |
| Gm14113 | predicted gene 14113 |  | 74 | 4.39 | 7.65 |
| Gm14114 | predicted gene 14114 |  | 7 | 3.71 | ND |
| Gm14770 | predicted gene 14770 |  | 7 | 4.53 | 5.21 |
| Gm14776 | predicted gene 14776 |  | 24 | 5.75 | 5.40 |
| Gm14808 | predicted gene 14808 |  | 10 | 4.61 | 4.08 |
| Gm14817 | predicted gene 14817 |  | 8 | 3.88 | 2.67 |
| Gm15222 | predicted gene 15222 |  | 18 | 3.89 | 2.72 |
| Gm15270 | predicted gene 15270 |  | 85 | 3.58 | 2.12 |
| Gm15326 | predicted gene 15326 |  | 13 | 2.00 | 4.27 |
| Gm15327 | predicted gene 15327 |  | 21 | 2.35 | 2.75 |
| Gm15535 | predicted gene 15535 |  | 15 | 3.94 | 3.64 |
| Gm15802 | predicted gene 15802 |  | 13 | 3.90 | 5.37 |
| Gm15834 | predicted gene 15834 |  | 24 | 2.29 | 2.60 |
| Gm15941 | predicted gene 15941 |  | 15 | 3.60 | 2.49 |
| Gm15972 | predicted gene 15972 |  | 36 | 3.70 | 2.04 |
| Gm16054 | predicted gene 16054 |  | 5 | 3.55 | ND |
| Gm16062 | predicted gene 16062 |  | 32 | 2.32 | 3.12 |
| Gm16082 | predicted gene 16082 |  | 5 | 5.16 | ND |
| Gm16104 | predicted gene 16104 |  | 26 | 3.63 | 3.28 |
| Gm16139 | predicted gene 16139 |  | 6 | 3.50 | 3.82 |
| Gm20619 | predicted gene 20619 |  | 10 | 2.04 | 5.08 |
| Gm2115 | predicted gene 2115 |  | 2372 | 6.65 | 7.76 |
| Gm2164 | predicted gene 2164 |  | 12 | 4.89 | 6.99 |
| Gm27202 | predicted gene 27202 |  | 106 | 7.88 | 3.03 |
| Gm27217 | predicted gene 27217 |  | 27 | 4.28 | 6.32 |
| Gm28177 | predicted gene 28177 |  | 14 | 4.63 | 2.99 |
| Gm29539 | predicted gene 29539 |  | 12 | 3.07 | 6.98 |
| Gm4128 | predicted gene 4128 |  | 10 | 2.18 | ND |
| Gm4189 | predicted gene 4189 |  | 21 | 2.60 | 2.22 |
| Gm4221 | predicted gene 4221 |  | 27 | 2.13 | 2.88 |
| Gm42463 | predicted gene 42463 |  | 15 | 2.31 | 3.46 |
| Gm42466 | predicted gene 42466 |  | 42 | 2.38 | 2.96 |
| Gm42683 | predicted gene 42683 |  | 24 | 2.47 | 3.95 |
| Gm42735 | predicted gene 42735 |  | 40 | 2.65 | 2.07 |
| Gm42788 | predicted gene 42788 |  | 67 | 3.21 | 5.66 |
| Gm42825 | predicted gene 42825 |  | 52 | 7.45 | ND |
| Gm42909 | predicted gene 42909 |  | 18 | 2.51 | 5.82 |
| Gm42942 | predicted gene 42942 |  | 11 | 2.14 | 2.52 |
| Gm42946 | predicted gene 42946 |  | 59 | 3.80 | 7.15 |
| Gm43084 | predicted gene 43084 |  | 8 | 3.51 | 6.43 |
| Gm43526 | predicted gene 43526 |  | 25 | 3.93 | 5.50 |
| Gm43527 | predicted gene 43527 |  | 43 | 3.23 | 5.89 |
| Gm43528 | predicted gene 43528 |  | 50 | 3.33 | 5.44 |
| Gm43560 | predicted gene 43560 |  | 79 | 2.32 | 2.17 |
| Gm43594 | predicted gene 43594 |  | 10 | 2.72 | ND |
| Gm43652 | predicted gene 43652 |  | 21 | 3.40 | 3.84 |
| Gm4419 | predicted gene 4419 |  | 19 | 2.61 | 2.05 |
| Gm44750 | predicted gene 44750 |  | 16 | 4.10 | 5.27 |
| Gm44883 | predicted gene 44883 |  | 23 | 2.32 | 4.35 |
| Gm44894 | predicted gene 44894 |  | 8 | 3.14 | 4.06 |
| Gm44895 | predicted gene 44895 |  | 16 | 4.64 | ND |
| Gm44897 | predicted gene 44897 |  | 18 | 3.99 | ND |
| Gm44898 | predicted gene 44898 |  | 8 | 3.83 | 6.22 |
| Gm45174 | predicted gene 45174 |  | 36 | 5.32 | ND |
| Gm4524 | predicted gene 4524 |  | 41 | 3.74 | 3.38 |
| Gm45393 | predicted gene 45393 |  | 10 | 4.81 | 3.46 |
| Gm45394 | predicted gene 45394 |  | 23 | 3.49 | 5.46 |
| Gm45620 | predicted gene 45620 |  | 11 | 6.16 | 3.16 |
| Gm45731 | predicted gene 45731 |  | 29 | 2.10 | 2.46 |
| Gm45869 | predicted gene 45869 |  | 36 | 2.56 | 5.81 |
| Gm4739 | predicted gene 4739 |  | 212 | 2.92 | 2.99 |
| Gm5454 | predicted gene 5454 |  | 124 | 4.85 | 2.15 |
| Gm5914 | predicted gene 5914 |  | 124 | 3.84 | 2.82 |
| Gm7537 | predicted gene 7537 |  | 12 | 2.86 | 6.90 |
| Gm807 | predicted gene 807 |  | 10 | 2.54 | 2.82 |
| Gm8495 | predicted gene 8495 |  | 11 | 3.01 | 2.56 |
| Gm9085 | predicted gene 9085 |  | 10 | 2.08 | 2.68 |
| Gm9930 | predicted gene 9930 |  | 13 | 2.29 | 3.09 |
| Gm9945 | predicted gene 9945 |  | 8 | 3.01 | 2.41 |
| Gm17308 | predicted gene, 17308 |  | 25 | 3.60 | 7.19 |
| Gm19196 | predicted gene, 19196 |  | 18 | 2.94 | 2.16 |
| Gm19445 | predicted gene, 19445 |  | 30 | 6.77 | 3.75 |
| Gm19514 | predicted gene, 19514 |  | 33 | 2.83 | 4.56 |
| Gm19554 | predicted gene, 19554 |  | 55 | 4.32 | 6.85 |
| Gm19744 | predicted gene, 19744 |  | 14 | 2.66 | 3.76 |
| Gm19935 | predicted gene, 19935 |  | 13 | 5.06 | 4.37 |
| Gm20172 | predicted gene, 20172 |  | 7 | 4.56 | 5.19 |
| Gm20754 | predicted gene, 20754 |  | 193 | 7.07 | 8.65 |
| Gm24784 | predicted gene, 24784 |  | 7 | 6.01 | ND |
| Gm25188 | predicted gene, 25188 |  | 31 | 3.72 | 3.37 |
| Gm26519 | predicted gene, 26519 |  | 7 | 4.10 | ND |
| Gm26660 | predicted gene, 26660 |  | 49 | 2.33 | 2.20 |
| Gm26674 | predicted gene, 26674 |  | 78 | 2.01 | 2.39 |
| Gm26728 | predicted gene, 26728 |  | 25 | 2.70 | 2.46 |
| Gm26797 | predicted gene, 26797 |  | 22 | 2.44 | 3.54 |
| Gm26930 | predicted gene, 26930 |  | 17 | 2.43 | 2.01 |
| Gm27011 | predicted gene, 27011 |  | 13 | 2.52 | 2.89 |
| Gm30177 | predicted gene, 30177 |  | 6 | 3.44 | ND |
| Gm32031 | predicted gene, 32031 |  | 128 | 3.00 | 2.23 |
| Gm32369 | predicted gene, 32369 |  | 6 | 2.72 | 3.33 |
| Gm32834 | predicted gene, 32834 |  | 11 | 3.54 | 2.71 |
| Gm32842 | predicted gene, 32842 |  | 11 | 3.91 | 2.16 |
| Gm33533 | predicted gene, 33533 |  | 6 | 4.39 | 5.69 |
| Gm33782 | predicted gene, 33782 |  | 16 | 4.22 | 5.85 |
| Gm33979 | predicted gene, 33979 |  | 33 | 5.02 | ND |
| Gm34777 | predicted gene, 34777 |  | 13 | 4.72 | 2.71 |
| Gm36939 | predicted gene, 36939 |  | 6 | 5.23 | ND |
| Gm36944 | predicted gene, 36944 |  | 396 | 5.82 | 6.08 |
| Gm36952 | predicted gene, 36952 |  | 12 | 3.01 | ND |
| Gm36988 | predicted gene, 36988 |  | 94 | 4.01 | 2.59 |
| Gm37056 | predicted gene, 37056 |  | 11 | 3.28 | 5.42 |
| Gm37181 | predicted gene, 37181 |  | 80 | 4.77 | 6.70 |
| Gm37211 | predicted gene, 37211 |  | 13 | 2.88 | 4.14 |
| Gm37331 | predicted gene, 37331 |  | 11 | 2.18 | 5.48 |
| Gm37419 | predicted gene, 37419 |  | 42 | 2.30 | 2.64 |
| Gm37443 | predicted gene, 37443 |  | 9 | 3.50 | 4.53 |
| Gm37459 | predicted gene, 37459 |  | 22 | 2.59 | 4.32 |
| Gm37526 | predicted gene, 37526 |  | 9 | 3.04 | 3.78 |
| Gm37602 | predicted gene, 37602 |  | 21 | 3.65 | 7.82 |
| Gm37626 | predicted gene, 37626 |  | 63 | 2.21 | 2.28 |
| Gm37725 | predicted gene, 37725 |  | 82 | 5.53 | 9.89 |
| Gm37767 | predicted gene, 37767 |  | 8 | 3.32 | 2.58 |
| Gm37855 | predicted gene, 37855 |  | 14 | 2.84 | 2.51 |
| Gm37880 | predicted gene, 37880 |  | 12 | 2.65 | 5.19 |
| Gm37965 | predicted gene, 37965 |  | 7 | 3.92 | 2.04 |
| Gm38031 | predicted gene, 38031 |  | 19 | 3.73 | 7.65 |
| Gm38243 | predicted gene, 38243 |  | 9 | 2.68 | 3.99 |
| Gm38255 | predicted gene, 38255 |  | 70 | 5.78 | 5.03 |
| Gm38260 | predicted gene, 38260 |  | 21 | 3.11 | 5.10 |
| Gm38335 | predicted gene, 38335 |  | 25 | 2.30 | 2.43 |
| Gm38353 | predicted gene, 38353 |  | 8 | 3.57 | ND |
| Gm39473 | predicted gene, 39473 |  | 15 | 6.98 | 3.96 |
| Gm42067 | predicted gene, 42067 |  | 35 | 2.80 | 2.27 |
| Gm43965 | predicted gene, 43965 |  | 14 | 4.04 | 2.98 |
| Gm44190 | predicted gene, 44190 |  | 29 | 2.60 | 2.26 |
| Gm44386 | predicted gene, 44386 |  | 32 | 2.35 | 2.43 |
| Gm44436 | predicted gene, 44436 |  | 62 | 5.29 | 8.20 |
| Gm44439 | predicted gene, 44439 |  | 179 | 5.19 | 9.72 |
| Gm44440 | predicted gene, 44440 |  | 77 | 4.39 | 5.50 |
| Gm44441 | predicted gene, 44441 |  | 44 | 3.64 | 7.99 |
| Gm46212 | predicted gene, 46212 |  | 26 | 2.37 | 2.02 |
| Gm46404 | predicted gene, 46404 |  | 22 | 2.42 | 2.49 |
| Gm47017 | predicted gene, 47017 |  | 52 | 5.66 | 6.03 |
| Gm47018 | predicted gene, 47018 |  | 28 | 5.79 | 8.19 |
| Gm47022 | predicted gene, 47022 |  | 31 | 3.44 | 7.28 |
| Gm47023 | predicted gene, 47023 |  | 7 | 3.65 | 3.60 |
| Gm47076 | predicted gene, 47076 |  | 16 | 2.60 | 2.28 |
| Gm47359 | predicted gene, 47359 |  | 13 | 4.49 | ND |
| Gm47547 | predicted gene, 47547 |  | 7 | 2.99 | 3.05 |
| Gm47591 | predicted gene, 47591 |  | 16 | 3.34 | 6.56 |
| Gm47592 | predicted gene, 47592 |  | 20 | 4.29 | 5.84 |
| Gm47621 | predicted gene, 47621 |  | 155 | 5.24 | 3.52 |
| Gm47623 | predicted gene, 47623 |  | 106 | 7.36 | 3.67 |
| Gm47624 | predicted gene, 47624 |  | 116 | 6.55 | 4.39 |
| Gm47700 | predicted gene, 47700 |  | 17 | 2.87 | ND |
| Gm47702 | predicted gene, 47702 |  | 41 | 6.26 | 6.62 |
| Gm47704 | predicted gene, 47704 |  | 19 | 2.75 | 4.32 |
| Gm47772 | predicted gene, 47772 |  | 19 | 3.30 | 3.49 |
| Gm47817 | predicted gene, 47817 |  | 143 | 2.11 | 2.19 |
| Gm47990 | predicted gene, 47990 |  | 90 | ND | 7.87 |
| Gm47991 | predicted gene, 47991 |  | 8 | ND | ND |
| Gm48259 | predicted gene, 48259 |  | 12 | 6.36 | 4.84 |
| Gm48261 | predicted gene, 48261 |  | 15 | 2.95 | 6.00 |
| Gm48427 | predicted gene, 48427 |  | 25 | 3.27 | 3.38 |
| Gm48497 | predicted gene, 48497 |  | 23 | 7.27 | ND |
| Gm48751 | predicted gene, 48751 |  | 18 | 3.53 | 2.84 |
| Gm4798 | predicted pseudogene 4798 |  | 30 | 2.25 | 2.09 |
| Gm5473 | predicted pseudogene 5473 |  | 8 | 2.75 | 3.26 |
| Gm6525 | predicted pseudogene 6525 |  | 31 | 3.98 | 2.73 |
| Prnp | prion protein | g,s | 5306 | 2.31 | 2.79 |
| Prima1 | proline rich membrane anchor 1 |  | 852 | 6.63 | 8.21 |
| Psrc1 | proline/serine-rich coiled-coil 1 |  | 38 | 2.58 | 3.10 |
| Prrt1 | proline-rich transmembrane protein 1 |  | 169 | 4.77 | 3.29 |
| Psapl1 | prosaposin-like 1 |  | 9 | 3.83 | 4.49 |
| Ppp1r14c | protein phosphatase 1, regulatory inhibitor subunit 14C |  | 540 | 4.65 | 6.13 |
| Ppp1r1b | protein phosphatase 1, regulatory inhibitor subunit 1B | s | 104 | 4.43 | 4.81 |
| Ppp1r26 | protein phosphatase 1, regulatory subunit 26 |  | 74 | 2.34 | 2.75 |
| Ppp2r2b | protein phosphatase 2, regulatory subunit B, beta |  | 319 | 2.30 | 4.26 |
| Ptprz1 | protein tyrosine phosphatase, receptor type Z, polypeptide 1 | g | 5121 | 6.21 | 7.29 |
| Ptprd | protein tyrosine phosphatase, receptor type, D | s | 1071 | 2.22 | 3.63 |
| Plp1 | proteolipid protein (myelin) 1 | g,s | 5346 | 3.14 | 5.81 |
| Pcdh10 | protocadherin 10 |  | 166 | 2.07 | 2.92 |
| Pcdhb10 | protocadherin beta 10 |  | 48 | 3.85 | 3.26 |
| Pcdhb8 | protocadherin beta 8 |  | 30 | 2.64 | 2.85 |
| P2ry12 | purinergic receptor P2Y, G-protein coupled 12 | g,p | 274 | 3.70 | 6.14 |
| Qrfpr | pyroglutamylated RFamide peptide receptor |  | 9 | 3.93 | 6.28 |
| Rab27b | RAB27B, member RAS oncogene family |  | 74 | 2.65 | 3.77 |
| Rab31 | RAB31, member RAS oncogene family |  | 1717 | 2.22 | 2.26 |
| Rgl3 | ral guanine nucleotide dissociation stimulator-like 3 |  | 37 | 2.38 | 2.63 |
| Rasgef1c | RasGEF domain family, member 1C |  | 1619 | 6.19 | 7.49 |
| Rit2 | Ras-like without CAAX 2 | s | 19 | 4.35 | 7.67 |
| Rbpjl | recombination signal binding protein for immunoglobulin kappa J region-like |  | 95 | 2.51 | 4.25 |
| Rflna | refilin A |  | 155 | 2.66 | 3.20 |
| Rfx4 | regulatory factor X, 4 (NDluences HLA class II expression) |  | 20 | 2.86 | 3.81 |
| Rlbp1 | retinaldehyde binding protein 1 |  | 33 | 3.12 | 5.69 |
| Rxrg | retinoid X receptor gamma | g | 764 | 5.70 | 6.79 |
| Arhgef16 | Rho guanine nucleotide exchange factor (GEF) 16 |  | 401 | 5.61 | 4.27 |
| Arhgef19 | Rho guanine nucleotide exchange factor (GEF) 19 |  | 164 | 3.44 | 5.09 |
| Arhgef26 | Rho guanine nucleotide exchange factor (GEF) 26 |  | 572 | 4.06 | 2.92 |
| Rtkn2 | rhotekin 2 |  | 30 | 3.07 | 2.08 |
| 1110032F04Rik | RIKEN cDNA 1110032F04 gene |  | 31 | 5.14 | 4.91 |
| 1500026H17Rik | RIKEN cDNA 1500026H17 gene |  | 36 | 3.62 | 3.63 |
| 1700010I14Rik | RIKEN cDNA 1700010I14 gene |  | 16 | 2.42 | 2.46 |
| 1700047M11Rik | RIKEN cDNA 1700047M11 gene |  | 239 | 4.81 | 5.26 |
| 1700057H15Rik | RIKEN cDNA 1700057H15 gene |  | 11 | 3.64 | ND |
| 1810010H24Rik | RIKEN cDNA 1810010H24 gene |  | 94 | 2.05 | 3.61 |
| 1810024B03Rik | RIKEN cDNA 1810024B03 gene |  | 135 | 2.30 | 2.25 |
| 2010204K13Rik | RIKEN cDNA 2010204K13 gene |  | 48 | 3.01 | 3.92 |
| 2010320O07Rik | RIKEN cDNA 2010320O07 gene |  | 19 | 3.51 | 5.43 |
| 2310016G11Rik | RIKEN cDNA 2310016G11 gene |  | 7 | 2.45 | 5.27 |
| 2610020C07Rik | RIKEN cDNA 2610020C07 gene |  | 66 | 2.44 | 2.57 |
| 2900002M20Rik | RIKEN cDNA 2900002M20 gene |  | 6 | 4.66 | ND |
| 2900022M07Rik | RIKEN cDNA 2900022M07 gene |  | 33 | 4.53 | 6.28 |
| 2900052L18Rik | RIKEN cDNA 2900052L18 gene |  | 33 | 2.33 | 2.69 |
| 3110009E18Rik | RIKEN cDNA 3110009E18 gene |  | 80 | 2.11 | 2.59 |
| 3110021N24Rik | RIKEN cDNA 3110021N24 gene |  | 17 | 2.23 | 2.40 |
| 3110080E11Rik | RIKEN cDNA 3110080E11 gene |  | 113 | 4.07 | 7.02 |
| 4632428C04Rik | RIKEN cDNA 4632428C04 gene |  | 41 | 3.44 | 2.85 |
| 4732491K20Rik | RIKEN cDNA 4732491K20 gene |  | 92 | 3.00 | 3.74 |
| 4930469K13Rik | RIKEN cDNA 4930469K13 gene |  | 120 | 3.99 | 8.56 |
| 4930480K15Rik | RIKEN cDNA 4930480K15 gene |  | 21 | 3.90 | 7.77 |
| 4930505M18Rik | RIKEN cDNA 4930505M18 gene |  | 12 | 2.92 | 5.81 |
| 4930509J09Rik | RIKEN cDNA 4930509J09 gene |  | 11 | 2.80 | 5.31 |
| 4930570D08Rik | RIKEN cDNA 4930570D08 gene |  | 26 | ND | 5.70 |
| 4930570G19Rik | RIKEN cDNA 4930570G19 gene |  | 44 | 2.25 | 3.98 |
| 4930579J19Rik | RIKEN cDNA 4930579J19 gene |  | 31 | 3.10 | 2.10 |
| 4930579K19Rik | RIKEN cDNA 4930579K19 gene |  | 8 | 2.94 | 3.05 |
| 4930589L23Rik | RIKEN cDNA 4930589L23 gene |  | 25 | 2.23 | 2.66 |
| 4932435O22Rik | RIKEN cDNA 4932435O22 gene |  | 17 | 2.83 | 3.02 |
| 4933407E24Rik | RIKEN cDNA 4933407E24 gene |  | 11 | 2.64 | 5.56 |
| 4933407I08Rik | RIKEN cDNA 4933407I08 gene |  | 16 | 5.55 | 6.20 |
| 5330409N07Rik | RIKEN cDNA 5330409N07 gene |  | 11 | 2.26 | 4.25 |
| 5430427N15Rik | RIKEN cDNA 5430427N15 gene |  | 6 | 3.16 | 2.95 |
| 5430435K18Rik | RIKEN cDNA 5430435K18 gene |  | 15 | 5.93 | 7.02 |
| 5930430L01Rik | RIKEN cDNA 5930430L01 gene |  | 94 | 3.45 | 2.24 |
| 6030407O03Rik | RIKEN cDNA 6030407O03 gene |  | 12 | 3.40 | 3.40 |
| 6330403L08Rik | RIKEN cDNA 6330403L08 gene |  | 409 | 3.77 | 2.84 |
| 6430503K07Rik | RIKEN cDNA 6430503K07 gene |  | 38 | 5.09 | 6.85 |
| 8030445P17Rik | RIKEN cDNA 8030445P17 gene |  | 29 | 3.48 | 6.65 |
| 9230112E08Rik | RIKEN cDNA 9230112E08 gene |  | 115 | 2.10 | 2.23 |
| 9330159F19Rik | RIKEN cDNA 9330159F19 gene |  | 144 | 3.93 | 5.20 |
| 9430041J12Rik | RIKEN cDNA 9430041J12 gene |  | 50 | 4.01 | 7.47 |
| 9630001P10Rik | RIKEN cDNA 9630001P10 gene |  | 40 | 4.07 | 5.49 |
| A130050O07Rik | RIKEN cDNA A130050O07 gene |  | 70 | 2.80 | 3.14 |
| A230081H15Rik | Riken cDNA A230081H15 gene |  | 39 | 3.43 | 6.20 |
| A330058E17Rik | RIKEN cDNA A330058E17 gene |  | 15 | 2.04 | 2.86 |
| A530095I07Rik | RIKEN cDNA A530095I07 gene |  | 10 | 2.66 | 5.15 |
| A930018P22Rik | RIKEN cDNA A930018P22 gene |  | 13 | 5.27 | 6.22 |
| B230312C02Rik | RIKEN cDNA B230312C02 gene |  | 25 | 2.13 | 2.70 |
| B230317F23Rik | RIKEN cDNA B230317F23 gene |  | 53 | 2.15 | 3.56 |
| B230359F08Rik | RIKEN cDNA B230359F08 gene |  | 7 | 3.29 | ND |
| B630019A10Rik | RIKEN cDNA B630019A10 gene |  | 19 | 3.07 | 2.63 |
| C030006N10Rik | RIKEN cDNA C030006N10 gene |  | 48 | 3.73 | 7.19 |
| C030029H02Rik | RIKEN cDNA C030029H02 gene |  | 13 | 3.87 | 5.51 |
| C130071C03Rik | RIKEN cDNA C130071C03 gene |  | 48 | 2.89 | 7.96 |
| C230035I16Rik | RIKEN cDNA C230035I16 gene |  | 18 | 2.97 | 2.74 |
| C530008M17Rik | RIKEN cDNA C530008M17 gene |  | 153 | 2.73 | 4.04 |
| D030047H15Rik | RIKEN cDNA D030047H15 gene |  | 10 | 2.88 | 2.62 |
| D030068K23Rik | RIKEN cDNA D030068K23 gene |  | 248 | 6.28 | 7.43 |
| D930032P07Rik | RIKEN cDNA D930032P07 gene |  | 19 | 2.91 | 4.15 |
| I0C0044D17Rik | RIKEN cDNA I0C0044D17 gene |  | 28 | 4.83 | 4.79 |
| Rnf219 | ring finger protein 219 |  | 188 | 2.41 | 2.17 |
| S100b | S100 protein, beta polypeptide, neural | g,p,s | 1788 | 3.12 | 5.34 |
| Scrg1 | scrapie responsive gene 1 |  | 62 | 5.00 | 6.18 |
| Sec14l2 | SEC14-like lipid binding 2 |  | 80 | 3.12 | 2.86 |
| Sfrp1 | secreted frizzled-related protein 1 |  | 1702 | 2.03 | 4.11 |
| Sfrp5 | secreted frizzled-related sequence protein 5 |  | 2689 | 3.25 | 4.09 |
| Sema3e | sema domain, immunoglobulin domain (Ig), short basic domain, secreted, (semaphorin) 3E | s | 744 | 2.12 | 7.11 |
| Stk32a | serine/threonine kinase 32A |  | 372 | 4.85 | 6.61 |
| Sh3gl3 | SH3-domain GRB2-like 3 | s | 215 | 3.48 | 2.94 |
| Shc4 | SHC (Src homology 2 domain containing) family, member 4 |  | 301 | 2.29 | 4.95 |
| Shisa2 | shisa family member 2 |  | 67 | 2.81 | 2.14 |
| Shisa4 | shisa family member 4 |  | 449 | 2.87 | 2.67 |
| Sgo1 | shugoshin 1 |  | 96 | 2.17 | 2.95 |
| Sppl2b | signal peptide peptidase like 2B |  | 512 | 2.42 | 2.24 |
| Ssbp1 | single-stranded DNA binding protein 1 |  | 666 | 2.49 | 2.49 |
| Slain1 | SLAIN motif family, member 1 |  | 27 | 2.04 | 5.33 |
| Slitrk1 | SLIT and NTRK-like family, member 1 | s | 452 | 6.40 | 6.56 |
| Slitrk3 | SLIT and NTRK-like family, member 3 | s | 879 | 7.68 | 7.87 |
| Slitrk5 | SLIT and NTRK-like family, member 5 | s | 92 | 4.09 | 5.06 |
| Svip | small VCP/p97-interacting protein |  | 374 | 3.42 | 3.58 |
| Soga3 | SOGA family member 3 |  | 24 | 2.42 | 5.44 |
| Slc13a5 | solute carrier family 13 (sodium-dependent citrate transporter), member 5 |  | 22 | 3.69 | ND |
| Slc22a17 | solute carrier family 22 (organic cation transporter), member 17 |  | 671 | 2.63 | 3.38 |
| Slc26a7 | solute carrier family 26, member 7 |  | 14 | 2.09 | ND |
| Slc27a6 | solute carrier family 27 (fatty acid transporter), member 6 |  | 51 | 2.81 | 4.27 |
| Slc35d3 | solute carrier family 35, member D3 |  | 58 | 4.17 | 5.35 |
| Slc35f1 | solute carrier family 35, member F1 |  | 1805 | 5.62 | 3.58 |
| Slc8a3 | solute carrier family 8 (sodium/calcium exchanger), member 3 | g,s | 211 | 4.52 | 5.54 |
| Sstr1 | somatostatin receptor 1 |  | 183 | 5.42 | 4.70 |
| Sorcs1 | sortilin-related VPS10 domain containing receptor 1 |  | 292 | 2.31 | 2.86 |
| Sorcs2 | sortilin-related VPS10 domain containing receptor 2 | s | 980 | 3.66 | 2.57 |
| Sowaha | sosondowah ankyrin repeat domain family member A |  | 54 | 2.02 | 3.15 |
| Sox2ot | SOX2 overlapping transcript (non-protein coding) |  | 51 | 2.05 | 4.36 |
| Sall1 | spalt like transcription factor 1 |  | 46 | 5.45 | 3.93 |
| Spon1 | spondin 1, (f-spondin) extracellular matrix protein |  | 1660 | 2.78 | 2.57 |
| Srcin1 | SRC kinase signaling inhibitor 1 | s | 155 | 4.54 | 4.86 |
| Sox10 | SRY (sex determining region Y)-box 10 | g | 3494 | 4.63 | 6.87 |
| Sox2 | SRY (sex determining region Y)-box 2 |  | 210 | 3.77 | 6.53 |
| Sox30 | SRY (sex determining region Y)-box 30 |  | 17 | 2.40 | 3.73 |
| Sox6 | SRY (sex determining region Y)-box 6 | g | 763 | 4.38 | 2.62 |
| Ss18l2 | SS18, nBAF chromatin remodeling complex subunit like 2 |  | 554 | 2.30 | 2.45 |
| St8sia1 | ST8 alpha-N-acetyl-neuraminide alpha-2,8-sialyltransferase 1 |  | 130 | 4.05 | 6.84 |
| St8sia2 | ST8 alpha-N-acetyl-neuraminide alpha-2,8-sialyltransferase 2 | g | 770 | 4.94 | 3.09 |
| Saxo2 | stabilizer of axonemal microtubules 2 |  | 22 | 2.28 | 2.24 |
| Stard10 | START domain containing 10 |  | 405 | 4.10 | 3.47 |
| Samd5 | sterile alpha motif domain containing 5 |  | 514 | 3.11 | 2.04 |
| Srd5a1 | steroid 5 alpha-reductase 1 |  | 256 | 2.99 | 3.26 |
| Sapcd1 | suppressor APC domain containing 1 |  | 7 | 2.81 | 2.82 |
| Sapcd2 | suppressor APC domain containing 2 |  | 29 | 2.15 | 3.17 |
| Syt9 | synaptotagmin IX |  | 91 | 3.15 | 6.09 |
| Tafa1 | TAFA chemokine like family member 1 |  | 49 | 3.42 | 5.84 |
| Tafa5 | TAFA chemokine like family member 5 |  | 842 | 4.77 | 5.92 |
| Tbx4 | T-box 4 |  | 31 | 2.04 | 2.45 |
| Tenm3 | teneurin transmembrane protein 3 |  | 556 | 2.69 | 2.74 |
| Tns3 | tensin 3 |  | 2573 | 3.18 | 3.12 |
| Tox | thymocyte selection-associated high mobility group box |  | 341 | 4.11 | 4.90 |
| Tmsb15l | thymosin beta 15b like |  | 14 | 3.43 | 4.74 |
| Tmsb15b1 | thymosin beta 15b1 |  | 29 | 3.64 | 3.59 |
| Tnik | TRAF2 and NCK interacting kinase |  | 546 | 2.92 | 3.18 |
| Tceal3 | transcription elongation factor A (SII)-like 3 |  | 54 | 3.03 | 2.94 |
| Tfap2a | transcription factor AP-2, alpha |  | 13 | 2.04 | 3.01 |
| Tagln3 | transgelin 3 |  | 26 | 4.54 | 3.35 |
| Tvp23bos | trans-golgi network vesicle protein 23B, opposite strand |  | 22 | 3.41 | 2.62 |
| Trpm3 | transient receptor potential cation channel, subfamily M, member 3 |  | 778 | 4.29 | 4.31 |
| Trpv3 | transient receptor potential cation channel, subfamily V, member 3 |  | 39 | 3.26 | 4.10 |
| Tram1l1 | translocation associated membrane protein 1-like 1 |  | 36 | 2.39 | 3.41 |
| Tmprss5 | transmembrane protease, serine 5 (spinesin) |  | 325 | 4.41 | 7.01 |
| Tmem121 | transmembrane protein 121 |  | 129 | 3.97 | 4.41 |
| Tmem196 | transmembrane protein 196 |  | 44 | 3.51 | 3.59 |
| Tmem200a | transmembrane protein 200A |  | 183 | 5.44 | 3.79 |
| Tmem26 | transmembrane protein 26 |  | 149 | 2.42 | 3.21 |
| Tmem88b | transmembrane protein 88B |  | 62 | 2.90 | 3.80 |
| Ttr | transthyretin |  | 7 | 3.62 | 3.05 |
| Trim2 | tripartite motif-containing 2 |  | 1415 | 2.52 | 2.64 |
| Tub | tubby bipartite transcription factor |  | 63 | 2.71 | 2.40 |
| Ttyh1 | tweety family member 1 |  | 812 | 4.47 | 4.69 |
| Tyrp1 | tyrosinase-related protein 1 |  | 131 | 2.07 | 7.17 |
| Usp51 | ubiquitin specific protease 51 |  | 13 | 2.99 | 3.49 |
| Ube2ql1 | ubiquitin-conjugating enzyme E2Q family-like 1 |  | 77 | 3.64 | 5.00 |
| Unc79 | unc-79 homolog |  | 122 | 2.90 | 5.90 |
| Unc80 | unc-80, NALCN activator |  | 2511 | 7.12 | 8.28 |
| Vxn | vexin |  | 93 | 4.86 | 5.55 |
| Vmn1r181 | vomeronasal 1 receptor 181 |  | 67 | 6.10 | 7.18 |
| Vstm2a | V-set and transmembrane domain containing 2A |  | 182 | 4.63 | 2.63 |
| Wdr31 | WD repeat domain 31 |  | 17 | 2.79 | 2.10 |
| Wnk3 | WNK lysine deficient protein kinase 3 |  | 20 | 2.36 | 3.16 |
| Wwc1 | WW, C2 and coiled-coil domain containing 1 | s | 92 | 2.38 | 4.62 |
| Xylt1 | xylosyltransferase 1 |  | 922 | 2.87 | 2.64 |
| Zfp114 | zinc finger protein 114 |  | 55 | 3.06 | 3.24 |
| Zfp428 | zinc finger protein 428 |  | 146 | 3.27 | 3.10 |
| Zfp536 | zinc finger protein 536 |  | 477 | 3.52 | 5.31 |
| Zfp811 | zinc finger protein 811 |  | 25 | 2.30 | 2.23 |
| Zcwpw1 | zinc finger, CW type with PWWP domain 1 |  | 145 | 2.26 | 3.39 |
| Zdbf2 | zinc finger, DBF-type containing 2 |  | 265 | 2.55 | 3.11 |
