# Supplementary material for: Specific labeling of synaptic schwann cells reveals unique cellular and molecular features
Source: eLife. 2020 Jun 25;9:e56935. doi: 10.7554/eLife.56935 (PMC7316509; doi:10.7554/eLife.56935)
Supplement: Supplementary file 2. [file elife-56935-supp2.docx]

**Supplementary File 2.** Primers used for cDNA preamplification and qPCR.

| Gene | Forward Primer (5’-3’) | Reverse Primer (5’-3’) |
| --- | --- | --- |
| 18S | GGACCAGAGCGAAAGCATTTG | GCCAGTCGGCATCGTTTATG |
| Ajap1 | ACAGCTTTTAGGACTCAGCTCCA | GATGGGAAGTCGACCGCAA |
| Bche | CTGCAGTAATTCCGAAATCAACA | GACCCTTCCGGTCTTGGTTG |
| Col20a1 | AGTCAGCCATACGGACACAT | CTCCAGGAAGTAGAGCCTCG |
| dsRed | TCCCAGCCCATAGTCTTCTTCT | GTGACCGTGACCCAGGACTC |
| Foxd3 | TCCATCCCCTCACTCACCTAA | CCCAGCGGACGGGTTGA |
| Gfp | AGAACGGCATCAAGGTGAACT | GGGGTGTTCTGCTGGTAGTG |
| Ncam1 | AAGAAAAGACTCTGGATGGGC | CAAGGAGGACACACGAGCAT |
| Nrxn1 | GGGCGACCAAGGTAAAAGTA | GCTGCTTTGAATGGGGTTTTGA |
| Pdgfa | GGTGGCCAAAGTGGAGTATGT | CTCACCTCACATCTGTCTCCTC |
| Pdlim4 | CTCACCATCTCGCGGGTTCA | AGATGATCGTGGCAGCCTTT |
